# Chronic Diseases Multimorbidity among Adult People Living with HIV at Hawassa University Comprehensive Specialized Hospital, Southern Ethiopia

**DOI:** 10.1155/2020/2190395

**Published:** 2020-01-22

**Authors:** Endrias Markos Woldesemayat

**Affiliations:** College of Medicine and Health Sciences, School of Public Health, Hawassa University, Ethiopia

## Abstract

**Background:**

Due to the wide implementation of antiretroviral therapy (ART), people living with HIV (PLWHIV) are now living longer. This increased the risk of developing noncommunicable chronic diseases (NCCDs) among them.

**Objective:**

We aimed to describe prevalence of NCCDs multimorbidity among PLWHIV at Hawassa University Comprehensive Specialized Hospital (HUCSH).

**Method:**

In April 2016, institution-based cross-sectional study was conducted among PLWHIV, aged ≥ 18 years at the ART unit of HUCSH. A nurse working in the ART unit interviewed patients and reviewed medical records. Data on the NCCDs and its risk factors were obtained. List of diseases considered in this study were arthritis, diabetes mellitus, hypertension, congestive heart failure (CHF), rheumatic heart diseases (RHD), chronic bronchitis, asthma, and cancer.

**Results:**

More than half of the respondents (196) had at least one of the NCCDs and 34 (8.9%) had multimorbidity. The main system of the body affected were the musculoskeletal system, 146 (38.2%) and respiratory system, 46 (12.0%). There was no significant difference in the prevalence of individual NCCDs by gender. Patients aged above 44 years, patients with ART duration of at least 6 years, and patients with higher CD4 counts had increased odds of having any one of the NCCDs. Multimorbidity patients with a longer ART duration had an increased risk.

**Conclusion:**

The prevalence of NCCD multimorbidity among PLWHIV was high. Monitoring the occurrence of NCCDs among PLWHIV and noncommunicable disease care is recommended.

## 1. Introduction

Due to an increased access and utilization of antiretroviral therapy (ART), people living with HIV (PLWHIV) are now living longer [1]. This in turn raised the risk of having noncommunicable chronic diseases (NCCDs) among them. Aging, the HIV itself, and the adverse effect of antiretroviral medications may escalate the risk of having NCCD morbidity [2–5]. Having NCCDs among the PLWHIV may deteriorate the quality of life and prognosis of HIV. Considering these facts, investigating the NCCD comorbidities and multimorbidity among PLWHIV has a public health importance [6, 7].

In 2016, the prevalence of HIV in Ethiopia was 1.1% [8]. In the other hand, in the study setting, a hospital-based study reported the prevalence of diabetes mellitus (DM) and hypertension (HTN) to be 12.2% and 10.5%, respectively [9]. In a retrospective cohort study conducted in South Ethiopia, the prevalence of NCCDs was 29.7% and this was in line with the WHO country profile report of 2014 [10, 11].

In HIV-negative people, the risk of having NCCDs and multimorbidity is higher among the elderly [1, 12, 13]. Diabetes mellitus, cardiovascular diseases like HTN, and chronic obstructive pulmonary diseases (COPDs) are among the most common comorbidities in patients living with HIV [14, 15]. Certain study reported, HIV-positive status is associated with an increased likelihood of having the NCCDs [16]. Increased age, immune suppression or advanced stage of AIDS, coexisting infections (viral and tuberculosis), social deprivation, longer duration with HIV infection, longer survival, and the traditional risk factors of NCCDs are among the factors associated with having NCCDs among HIV patients [17].

Having NCCDs decreases the functional status and quality of life of patients and it increases drug side effects, medical costs, disability, and mortality of patients in HIV-negative people [18–21]. There is an increased risk of NCCDs in PLWHIV [16], and the NCCD comorbidities increase with the HIV severity [4]. NCCD comorbidities would negatively affect the quality of life and the cost of patient care of PLWHIV [22–24]. Due to the combined effect of the HIV and the NCCDs, patients living with HIV may have a complex healthcare needs. An increasing number of comorbid conditions in PLWHIV is directly linked to an increase in the number of total medications [2]. NCCDs are the major causes of death among PLWHIV [17]. In countries like Ethiopia, multimorbidity associated with HIV could affect healthy aging and overwhelm the healthcare systems [25]. Investigating the NCCDs among PLWHIV is thus an important issue [7]. The investigator aimed to describe the prevalence of NCCDs multimorbidity among PLWHIV at Hawassa University Comprehensive Specialized Hospital (HUCSH). This is the first study to estimate the prevalence of NCCDs and multimorbidity among PLWHIV in the study area.

## 2. Methods

A cross-sectional institutional study was conducted among PLWHIV attending HUCSH. Hawassa town is the capital of both the Sidama Zone and the Southern Nations and Nationalities Peoples Region (SNNPR) in Ethiopia. The town is located at 275 Km to the South of Addis Ababa with a total population of more than 400,000. HUCSH is the largest hospital in southern Ethiopia with more than 300 beds. The hospital was established in 2004; and currently, it provides a comprehensive and specialized health services to peoples of the region and other peoples coming from the neighboring Oromia Region. The ART unit in the hospital started its service in June 2006.

The study was conducted in April 2016. During the study period, over 4,000 PLWHIV were under follow-up in the ART unit with a median duration of 6 years of follow-up. Of this, about 2,000 patients were taking ART. For this cross-sectional study, the study participants were recruited from adult patients (aged at least 18 years) taking ART in the hospital.

A questionnaire format was developed based on the objective of the study. Questions were first developed in English and translated into Amharic and then back translated to English. The investigator provided training to the data collector. Training included explanation of the study objectives and techniques for interviewing. A few days prior to the actual data collection, pretesting was done on 5% of the calculated sample size of PLWHIV. Based on the pretest, necessary amendments were done on the questionnaire. Having NCCDs was measured by an open question. Each study participant was interviewed after consulting the physician and taking his pills. Patient's cards were reviewed to obtain additional information from the history, physical examination, investigations, and the medications used. Weight and height of the patients were measured. A nurse working at the ART unit collected data. Supervision was done throughout the data collection process, and daily checking of the collected data was done and problems encountered were managed accordingly.

Chronic disease was defined as a nonreversible and noninfectious disease affecting specific body systems which was diagnosed by a physician. Presence of NCCDs was assessed mainly by interview, which was supplemented with the review of medical records. The lists of diseases considered in this study were rheumatoid arthritis, gouty arthritis, diabetes mellitus, HTN, congestive heart failure (CHF), rheumatic heart diseases (RHD), chronic bronchitis, asthma, and cancer in any of the body parts. Rheumatoid arthritis refers to a chronic, autoimmune inflammatory disease of the joint [26]. Gouty arthritis was defined as an inflammatory arthritis caused by the deposition of urate crystals in the joints, which occurs due to chronic hyperuricemia [27]. Physicians diagnosed arthritis based on clinical data (history, physical examination, and investigations) obtained from each patient. Diabetes mellitus was considered if a patient presented with episodes of hyperglycemia and glucose intolerance, as a result of lack of insulin or its defective action [28]. A participant having systolic blood pressure of 140 mmHg and/or diastolic blood pressure of 90 mmHg was considered hypertensive [29]. Asthma was defined as an incurable lung disease that causes breathing difficulties due to narrowing of the tubes that carry air to and from the lungs. Its symptoms is relieved with treatment [30]. Chronic bronchitis was defined as a condition characterized by bronchial hypersecretion, chronic cough, and sputum production [31]. A patient suffering from dyspnea, fatigue, and clinical signs of congestion was diagnosed as a case of congestive heart failure [32]. Rheumatic heart disease was considered if permanent damage to the valves of the heart was developed due to repeated episodes of acute rheumatic fever [33]. Study participants without symptom of the NCCDs described above because of taking medications for the conditions were also considered cases of the specific NCCD. Multimorbidity refers to the presence of at least two of the NCCDs.

Normal body mass index (BMI) considered in this study was 18.5–24.9 kg/m^2^. Social support refers to obtaining any kind of regular support from individuals or organizations, which was provided in kind or in the form of money. ART duration was defined as the time period measured since the initiation of the ARV medications up to the date of interview.

The outcome variables considered in this study were having any of the NCCDs and multimorbidity among PLWHIV. Explanatory variables determining having a NCCD or multimorbidity were sociodemographic variables (gender, age, literacy status, monthly household income) and other variables such as smoking, alcohol drinking, khat chewing, high BMI, lack of physical activity, and low consumption of fruits and vegetables. ART duration and the CD4 count level were clinical factors expected to determine the outcome measures.

Assuming a prevalence of multimorbidity of 65% [34], 95% confidence interval (CI), a precession of 5%, and adding 10% for possible nonresponse, the sample size required for this study was 380. We added 10% to what we have calculated. Every 3^rd^ PLWHIV who were on ART during the study period were interviewed until the required sample size was obtained.

The investigator entered and analyzed data using SPSS version 20 statistical packages (SPSS Inc., Chicago, IL, USA). BMI refers to the calculated value of an individual weight in kilogram divided by height in meter square. Age was grouped in to 18–29 years, 30–44 years, and >44 years, considering the availability of values in cells for each age group. Education was categorized into two values, people with no education and literates. People with no education are participants without formal education. Participants with any of the formal schooling were considered as literate. Exercise was defined as performing of at least 30 minutes of physical activity of moderate intensity for at least 4 days per week [12]. Insufficient consumption of fruits and vegetables refers to the consumption of less than 125 ml or 400 grams fruit and vegetable per day [35]. Smoking refers to the use of at least one cigarette per day for more than 6 months. Alcohol drinking was considered if the study participant previously or currently habitually consumed any amount of alcoholic beverage. Khat chewing refers to a habitual chewing of any amount of khat (a psychoactive substance).

Descriptive statistics was used to calculate and present the sociodemographic, behavioral, and clinical characteristics of the study participants. The difference in the prevalence of individual chronic diseases among men and women was evaluated. Logistic regression model was used to determine the association between outcome variables and the expected determinants. Variables with a *P* value of less than 0.2 in the bivariate analysis were included in the multivariate analysis. Odds ratio and a 95% confidence interval (CI) were used to describe the association between the dependent variables and the independent variables.

## 3. Results

All of the approached eligible PLWHIV participated in the study. The median (interquartile range (IQR)) age of the study participants was 35 (10) years. Majority of the study participants (235 (61.5%)) were female. Employed, married, and literate PLWHIV were 106 (27.7%), 215 (56.3%), and 339 (88.7%), respectively. The median (IQR) household income was 1,500 (2,000) Eth. Birr. More than three-fourth of the study participants had children and get social support. Details of the sociodemographic characteristics of the study participants are described in Table 1.

Study participants with a BMI score of at least 25 were 117 (30.6%). Majority (361 (94.5%)) of the patients had the habit of exercising. Alcohol drinking, khat chewing, and cigarette smoking were experienced by 185 (48.4%), 137 (35.9%), and 49 (12.8%) patients, respectively. Seven (1.8%) of the study participants responded as current alcohol drinkers. The median (IQR) duration on ART and CD4 counts of the study participants were 74 (57) months and 545.1 (314) cells/*μ*l, respectively (Table 2).

More than half (196 (51.3%)) of the respondents were affected by at least one of the NCCDs. While 34 (8.9%) had multimorbidity by these chronic diseases. Musculoskeletal problems like rheumatoid arthritis and gouty arthritis were reported by 146 (38.2%) of the study participants. Of which, the majority (143 (97.9%)) were affected by rheumatoid arthritis. The second leading system of the body affected was the respiratory system, the diseases being chronic bronchitis and asthma 46 (12.0%). Seventeen patients had cardiovascular problems; of this, HTN was reported by 16 (94.1%) of the patients. Malignant cancers affected 15 (8.2%) patients; of which, 8 (53.3%) were reproductive system cancers (Table 3). There was no significant difference in the prevalence of an individual chronic disease among men and women (Table 4).

In a bivariate analysis, factors like age above 44 years, ART duration, alcohol drinking, khat chewing, and CD4 count above 350 cells per *μ*l showed association with having at least one of the NCCDs. In a multivariate analysis, however, age above 44 years (adjusted odds ratio (AOR) = 2.5, 95% CI 1.3–4.9), ART duration of at least 6 years (AOR = 2.1, 95% CI 1.4–3.2), and CD4 count maintained the significance in predicting having a NCCD. Patients with a CD4 count of 200–350 cells per *μ*l (AOR = 2.7, 95% CI 1.1–6.7) and CD4 count of above 350 cells per *μ*l (AOR = 2.3, 95% CI 1.0–5.3) had an increased risk of having at least one of the NCCDs. Concerning multimorbidity, ART duration of at least six years showed statistically significant association both in a bivariate (AOR = 3.0, 95% CI 1.3–6.5) and in a multivariate analysis (AOR = 2.6, 95% CI 1.2–5.9). Tables 5 and 6 show factors associated with having NCCDs and NCCD multimorbidity.

## 4. Discussion

In this institutional-based cross-sectional study, high prevalence of NCCDs and high prevalence of multimorbidity among PLWHIV were observed. Arthritis was the predominant disease affected the study participants. There was no significant difference on the prevalence of NCCDs by gender. The risk of having any of the NCCD comorbidity was higher among patients above 44 years of age, patients with ART duration of at least 6 years, and patients with high CD4 count, while being on ART for at least six years was associated with an increased risk of NCCD multimorbidity.

A study in Brent UK reported that 29% of PLWHIV had one or more comorbidity [36]. In a population-based study in Canada, 34.4% of PLWHIV had at least one other physical condition [37]. In Tanzania, the proportion of PLWHIV with one or more NCCDs was 57.8% [38]. The prevalence of comorbidity among PLWHIV was 15.3% in Zimbabwe [39]. The current study finding was comparable to the report from Tanzania [38], but it was higher than the reports from the UK, Canada, and Zimbabwe [36, 37, 39]. The difference could be due to variation in the study design [37]. The current study is a facility-based study carried out on PLWHIV who were on ART at a tertiary health care unit, in which interview was used as a primary method of data collection for identifying chronic diseases diagnosed by physicians, which was supplemented with a record review. In population-based studies, usually low prevalence of chronic diseases is reported, because relatively more healthy people are involved in such studies. Moreover, participants in community-based studies may prefer responding to the absence of diseases and diagnosing NCCDs in such studies may not be easy and accurate. The method of diagnosis could be another explanation for the observed differences. In Brent England, they did a retrospective study based on a confirmed evidence by investigations [36]. In retrospective studies, patients who do not complain for the presence of symptoms of a chronic disease may not be investigated and thus they may not be diagnosed. This may lower the prevalence of NCCDs. A similar explanation may apply for the observed difference in the prevalence of NCCDs between the current study and the report from Zimbabwe [39].

The commonly observed individual NCCDs occurring among PLWHIV in Australia, London and Ontario, Canada were mental health problems, followed by cardiovascular diseases [36, 37, 40]. In Zimbabwe, HTN, asthma, type 2 diabetes mellitus, cancer, and congestive cardiac failure were among the leading diseases reported [39]. In the current study, the commonest reported disease was arthritis. This finding is comparable to the report from Tanzania, where arthritis was the leading NCD that affected PLWHIV [38].

The prevalence of multimorbidity in the current study was lower than the reports from Canada (65%) and Australia (54.5%) [34, 40]. The differences could be due to the difference in the prevalence of the risk factors. In Canada, almost two-third of participants were overweight (36%) or obese (29%) [34] and various studies confirmed that there is an increased risk of multimorbidity among people with high BMI [34, 41]. In the current study, only 30.4% of PLWHIV had a BMI score of over 24.9. The mean age of the study participants in the Australia study was 51.8 years [40]. Study participants in the current study were younger than the study participants in these settings. There is an increased risk of multimorbidity among older people [37, 41, 42]. The investigator suggests the importance of controlling the modifiable risk factors and follow-up of PLWHIV with the nonmodifiable risk factors of NCCDs to lower the burden.

Current smoking was reported by 12-23% of men and 1-3% of women in Tanzania and Uganda [43]. Similar measure was 14.7% in Cambodia [44]. Only 0.3% of the current study participants were current tobacco smoker and women smoked less than men. This was as expected and in agreement to the finding in another study [44]. Problem of drinking alcohol was 6-15% in Tanzania and Uganda [43] and 54.7% in Cambodia [44]. Only 1.8% of the study participants in the current study responded as current alcohol drunkers. This finding was lower than the reports in other settings [43, 44]. The low level of current tobacco smoking and alcohol drinking among the study participants in the current study could be related to the change in behavior of the PLWHIV after being diagnosed with the HIV and started the ART.

Gender was one of the risk factors of NCCDs among PLWHIV [17, 39]. Contrary to the reports in these studies, in the current study, there was no association between gender and having of any of the NCCDs or having of multimorbidity. Higher age categories were significantly associated with comorbidity risk in Zimbabwe [39]. In agreement to their finding, in the current study, an increase in age predicted the risk of having an individual chronic disease. However, our study did not show the statistical association between age and multimorbidity. In other settings, increasing age was the risk factor for the presence of multiple health problems [37, 41, 42]. The investigator suggests the importance of early identification and management of chronic diseases among the elderly PLWHIV.

Higher CD4 values increased the risk for multimorbidity [17, 45]. Contrary to this report, no association was observed between CD4 level and multi-morbidity in the current study. However, there was a higher risk of having at least one of the NCCDs among PLWHIV with higher CD4 count. Another study reported that increased immune suppression was among the determinants of NCCDs [17]. Continuous monitoring of PLWHIV for having of NCCDs and delivering appropriate intervention is important to reduce the burden of NCCD comorbidity among PLWHIV. Particular attention should be given to patients with higher CD4 level.

In the current study, PLWHIV with at least 6 years of ART had an increased risk of developing both an individual NCCD and multimorbidity. This is in agreement to a previous report [17]. Longer duration of exposure to ART, especially exposure to protease inhibitors, was reported to increase the risk of NCCDs among PLWHIV [17]. Screening of PLWHIV with longer duration on ART could help in timely identifying the NCCDs. This helps to provide appropriate intervention in order to limit the effect of the diseases.

Obesity was associated with an increased odds of multimorbidity [34] and the prevalence of multimorbidity increased with each progressive BMI category [41]. Higher levels of traditional NCCD risk factors among PLWHIV (which includes higher BMI) were also among the associated risk factors reported for having any one of the NCCDs [17]. In the current study, none of the lifestyle factors showed association with having of any one of the NCCDs or multimorbidity by the NCCDs. This could be due to the limitation in the study design used to measure associations. Analytical study design could have been used to investigate such associations.

## 5. Limitation of the Study

This study has limitations. The dataset used had missing values in some of the measured characteristics. However, the missing values were low (less than 2%). Therefore, missing values are less likely to affect conclusion of the study. The study design used in the current study may be inappropriate for measuring association between exposure factors and the outcome variables. A cohort study or a case control study design could have been used to determine such kind of causal relationships.

## 6. Conclusion

In conclusion, high prevalence of NCCDs and multimorbidity was observed among PLWHIV. The main chronic disease that affected the patients was arthritis. There was no significant difference in the occurrence of chronic diseases among men and women. Age, ART duration, and CD4 count were determinant factors of having any one of the NCCDs, while being on ART for at least for six years predicted having multimorbidity. Monitoring the occurrence of NCCDs among PLWHIV with the risk factors and noncommunicable diseases care among PLWHIV is recommended.

## Figures and Tables

**Table 1:** Sociodemographic characteristics of people living with HIV at HUCSH, April 2016.

Characteristics	Value	Number	%
Gender	Male	147	38.5
	Female	235	61.5

Age in years	Median (IQR)	35 (10)	

Occupation	Employed	106	27.7
	House wife	96	25.1
	Daily laborer	84	22.0
	Merchant	54	14.2
	No job	14	3.7
	Other	28	7.3

Marital status	Single	41	10.7
	Married	215	56.3
	Divorced	60	15.7
	Widowed	59	15.4
	Missing	7	1.8

Household income in Eth. Birr^∗^	Median (IQR)	1,500 (2,000)	

Educational status	No education	43	11.3
	Literate	339	88.7

Having children	Yes	294	77.0
	No	71	18.6
	Missing	17	4.5

Social support	Yes	305	79.8
	No	74	19.4
	Missing	3	0.8

Household income had 8 missing values; exchange rate during the study period was 1 USD to 21.4 Ethiopian Birr.

**Table 2:** Behavioral and clinical characteristics of people living with HIV at HUCSH, April 2016.

Characteristics	Value	Number	%
BMI	<25	265	69.4
	≥25	117	30.6

Exercise	Yes	361	94.5
	No	21	5.5

Fruits and vegetables consumption	Yes	376	98.4
	No	6	1.6

Alcohol drinking	Yes	185	48.4
	No	197	51.6

Khat chewing	Yes	137	35.9
	No	245	64.1

Cigarette smoking	Yes	49	12.8
	No	333	87.2

Duration of ART in months^∗^	Median (IQR)	74 (57)	

Most recent CD4 count (cells/*μ*l)	Median (IQR)	545.1 (314)	

ART: antiretroviral therapy; BMI: body mass index. Duration of ART in months has 4 missing values, CD4 count had 1 missing value.

**Table 3:** Prevalence of chronic diseases by systems of the body among people living with HIV at HUCSH, April 2016.

Body systems affected	Diseases	Cases (%)
Musculoskeletal system	Rheumatoid arthritis, gout	146 (38.2)
Respiratory system	Chronic bronchitis, asthma	46 (12.0)
Cardiovascular system	HTN, CHF, RHD	17 (4.5)
Any cancer	Cancer	15 (3.9)
Endocrine system	Diabetes mellitus	8 (2.1)
Chronic diseases	Any chronic diseases	196 (51.3)
Multimorbidity		34 (8.9)

HTN: hypertension; CHF: congestive heart failure; RHD: rheumatic heart disease.

**Table 4:** Difference in prevalence of chronic diseases by gender among people living with HIV at HUCSH, April 2016.

Chronic diseases	Value	Men, *n* (%)	Women, *n* (%)	Total	*P* value
Arthritis	Yes	55(37.4)	91 (38.7)	146 (38.2)	0.8
	No	92 (62.6)	144 (61.3)	236 (61.8)	

Chronic bronchitis	Yes	9 (6.1)	19 (8.1)	28 (7.3)	0.47
	No	138 (93.9)	216 (91.9)	354 (92.7)	

Bronchial asthma	Yes	6 (4.1)	16 (6.8)	22 (5.8)	0.27
	No	141 (95.9)	219 (93.2)	360 (94.2)	

Hypertension	Yes	7 (4.8)	9 (3.8)	16 (4.2)	0.7
	No	140 (95.2)	226 (96.2)	366 (95.8)	

Cancer	Yes	4 (2.7)	11 (4.7)	15 (3.9)	0.3
	No	143 (97.3)	231 (95.3)	374 (96.1)	

Diabetes mellitus	Yes	4 (2.7)	4 (1.7)	8 (2.1)	0.5
	No	143 (97.3)	231 (98.3)	374 (97.9)	

Chronic diseases	Yes	72 (49.0)	124 (52.8)	196 (51.3)	0.5
	No	75 (51.0)	111 (47.2)	186 (48.7)	

*n* = number of respondents for the category.

**Table 5:** Risk factors of having a chronic disease among people living with HIV at HUCSH, April 2016.

Variables	Value	Chronic diseases		COR (95% CI)	AOR (95% CI)
		Yes	No		
Age in years	18–29	39	54		
	30–44	107	107	1.4 (0.9 – 2.2)	1.3 (0.7 – 2.2)
	≥45	50	25	2.8 (1.5–5.2)	2.6 (1.3–5.0)

ART duration	<6 years	77	110		
	≥6 years	119	76	2.3 (1.5–3.4)	2.0 (1.3–3.2)

Social support^∗^	Yes	150	155		
	No	44	30	1.5 (0.9–2.5)	1.4 (0.8–2.5)

Fruit/vegetable consumption	Yes	195	181		
	No	1	5	0.2 (0.0–1.6)	0.2 (0.0–1.6)

Alcohol drinking	Yes	110	75	1.9 (1.2–2.8)	1.6 (0.9–2.9)
	No	86	111		

Khat chewing	Yes	82	55	1.7 (1.1–2.5)	1.1 (0.6–2.1)
	No	114	131		

BMI	<25	128	137		
	≥25	68	49	1.5 (0.9–2.4)	1.6 (0.9–2.6)

CD4 count (cells/*μ*l)^∗^	<200	10	21		
	200–350	36	33	2.3 (0.9–5.6)	2.7 (1.1–6.8)
	>350	149	132	2.4 (1.1–5.2)	2.3 (1.0–5.2)

COR: crude odds ratio; AOR: adjusted odds ratio; 95% CI: 95% confidence interval: BMI: body mass index. ^∗^The variables have missing values.

**Table 6:** Risk factors of multimorbidity among people living with HIV at HUCSH, April 2016.

Variables	Value	Chronic diseases		COR (95% CI)	AOR (95% CI)
		Yes	No		
Age in years	18–29	4	89		
	30–44	22	192	2.6 (0.9–7.9)	2.8 (0.9–8.7)
	≥45	8	67	2.7 (0.8–9.5)	2.3 (0.6–8.5)

ART duration	<6 years	9	178		
	≥6 years	25	170	3.0 (1.3–6.5)	2.6 (1.1–5.8)

Social support^∗^	Yes	23	282		
	No	10	64	1.9 (0.9–4.2)	2.0 (0.9–4.6)

Alcohol drinking	Yes	20	165	1.6 (0.8–3.2)	1.0 (0.4–2.8)
	No	14	183		

Khat chewing	Yes	16	121	1.7 (0.8–3.4)	1.1 (0.4–3.2)
	No	18	227		

Smoking	Yes	8	41	2.3 (0.9–5.3)	2.4 (0.9–6.5)
	No	26	307		

COR: crude odds ratio; AOR: adjusted odds ratio; 95% CI: 95% confidence interval; BMI: body mass index. ^∗^The variable has missing value.

## Data Availability

The datasets used and or analyzed during the current study are available from the author on reasonable request.
